# Supplementary material for: Case study of resecabtagene autoleucel in a subject with diffuse cutaneous systemic sclerosis treated in the RESET-SSc trial
Source: Mol Ther Adv. 2025 Dec 26;34(1):201663. doi: 10.1016/j.omta.2025.201663 (PMC13182784; doi:10.1016/j.omta.2025.201663)
Supplement: Document S1. Figures S1–S3 [file mmc1.pdf]

OMTA, Volume *34*

## Supplemental information

# Case study of resecabtagene autoleucel in a subject with diffuse cutaneous systemic sclerosis treated in the RESET-SSc trial

**Daniel Nunez, Jenell R. Volkov, Courtney Little, Monalisa Ghosh, Pei-Suen Tsou, Thomas Furmanak, Poulami Dey, Lam C. Tsoi, Carleigh Zahn, Rachael Bogle, Yuli Cai, Jennifer Fox, Jason Stadanlick, Mallorie Werner, Zachary Vorndran, Larissa Ishikawa, Alexandra Ellis, Jazmean Williams, Justin Cicarelli, Steve Flannagan, Danielle Kobulsky, Quynh Lam, Chris Schmitt, Fatemeh Nezhad, Daniel Thompson, Dominick Braccia, Tania Gonzalez Rivera, Raj Tummala, Johann E. Gudjonsson, Charles Ross, Gwendolyn Binder, David Chang, Samik Basu, and Dinesh Khanna**

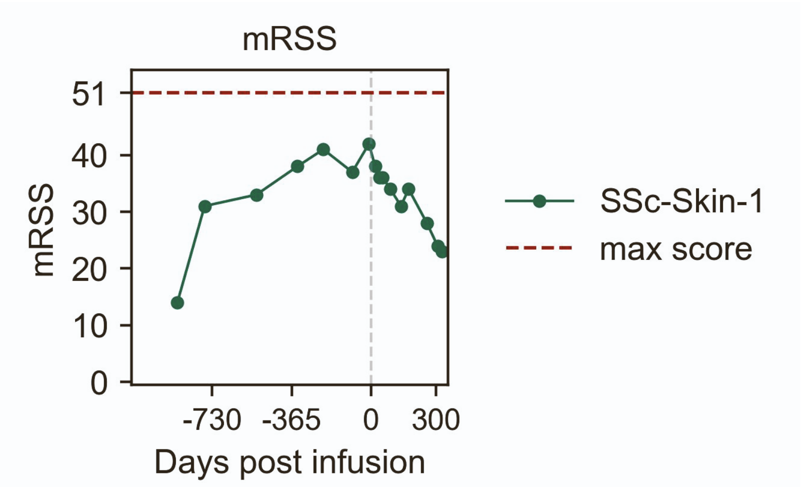


**Figure S1.** Historical mRSS from patient prior to rese-cel infusion and for up to 36 weeks post-infusion. Dashed vertical line represents infusion of rese-cel. X-axis is time from infusion. Red horizontal dashed line represents maximum possible mRSS.

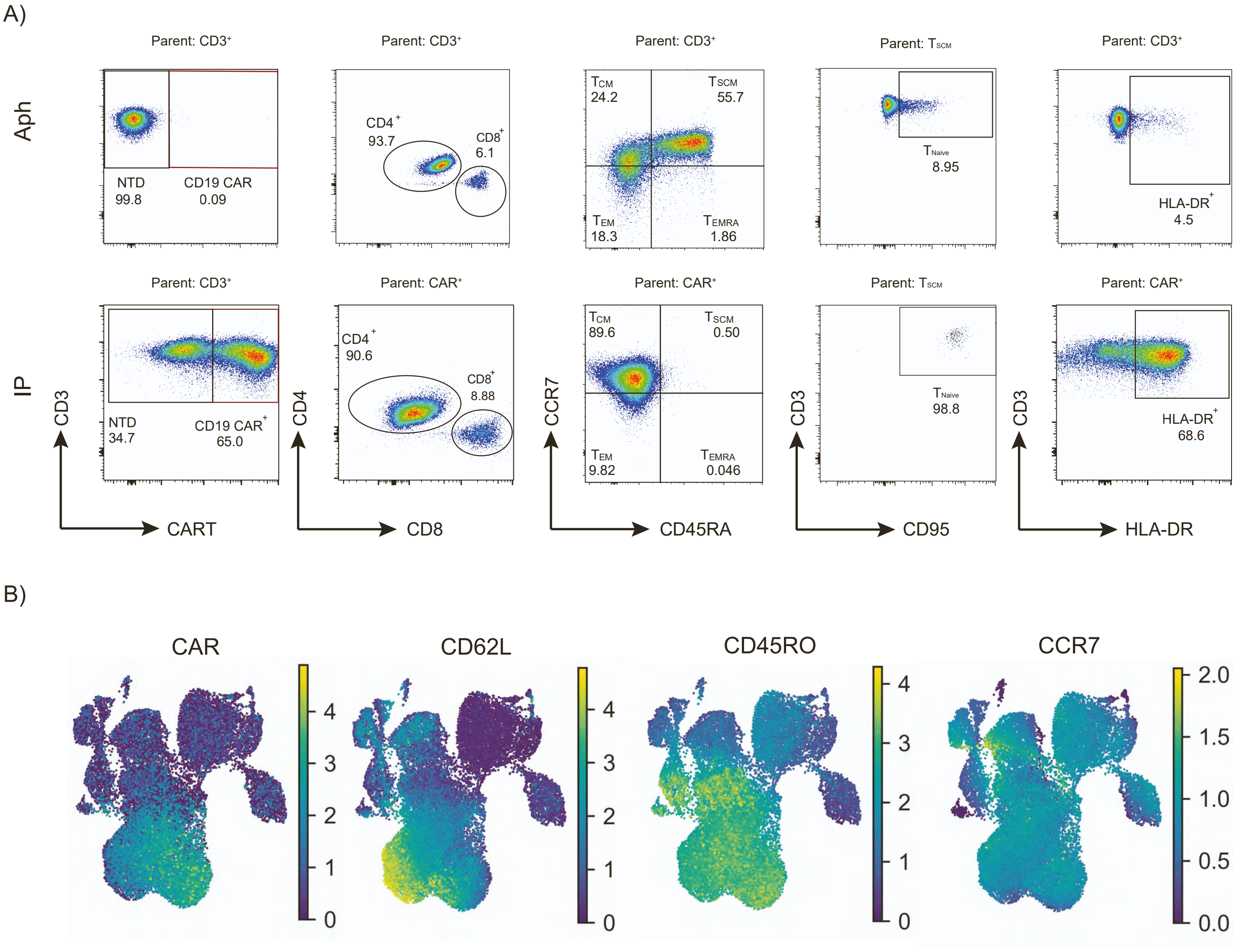


**Figure S2. (A)** Flow cytometry plots from patient's apheresis (Aph) and infusion product (IP). From left to right: the percentage of $CD3^+$ T cells that are $CAR^+$, the prorption of T cells (for Aph) and of $CAR^+$ T cells (for IP) that are $CD4^+$ and $CD8^+$, the memory phenotype of T cells (for Aph) and of $CAR^+$ T cells (for IP) via CCR7 and CD45RA labeling, the CD95 expression within the $T_{SCM}$ parent gate, and the percentage HLA-$DR^+$ T cells (for Aph) and of $CAR^+$ T cells (for IP). **(B)** Single-cell sequencing from patient's Aph and IP. CAR expression is shown as the natural logarithm of the CAR mRNA count divided by the total RNA counts per cell. CD62L, CD45RO, and CCR7 expression is shown as the natural logarithm of each protein count divided by the total portein counts per cell.

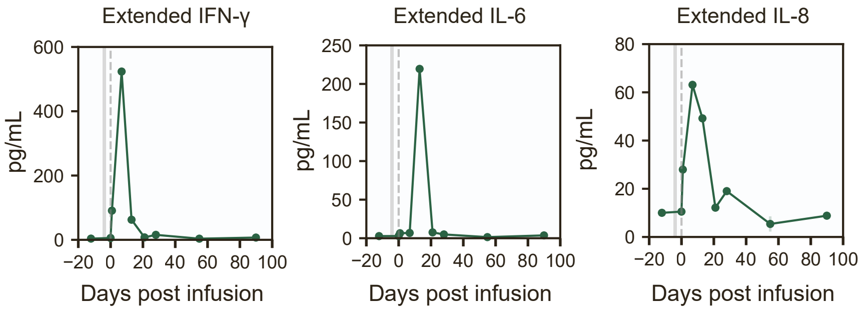


**Figure S3.** Extended serum cytokine profiling from patient SSc-Skin-1 over the first 12 weeks following rese-cel infusion. From left to right, plots depict serum concentrations of IFN-γ, IL-6, and IL-8 in pg/mL. The gray vertical dotted line indicates the day of infusion and the gray vertical shading indicates the period in time when the patient underwent preconditioning.
