# Supplementary material for: Case study of resecabtagene autoleucel in a subject with diffuse cutaneous systemic sclerosis treated in the RESET-SSc trial
Source: Mol Ther Adv. 2025 Dec 26;34(1):201663. doi: 10.1016/j.omta.2025.201663 (PMC13182784; doi:10.1016/j.omta.2025.201663)
Supplement: Document S2. Article plus supplemental information [file mmc2.pdf]

Molecular Therapy
Advances
Original Article

Case study of resecabtagene autoleucel in a subject with diffuse cutaneous systemic sclerosis treated in the RESET-SSc trial

Daniel Nunez,[1,3] Jenell R. Volkov,[1,3] Courtney Little,[1,3] Monalisa Ghosh,[2,3] Pei-Suen Tsou,[2,3] Thomas Furmanak,[1] Poulami Dey,[2] Lam C. Tsoi,[2] Carleigh Zahn,[2] Rachael Bogle,[2] Yuli Cai,[2] Jennifer Fox,[2] Jason Stadanlick,[1] Mallorie Werner,[1] Zachary Vorndran,[1] Larissa Ishikawa,[1] Alexandra Ellis,[1] Jazmean Williams,[1] Justin Cicarelli,[1] Steve Flannagan,[1] Danielle Kobulsky,[1] Quynh Lam,[1] Chris Schmitt,[1] Fatemeh Nezhad,[1] Daniel Thompson,[1] Dominick Braccia,[1] Tania Gonzalez Rivera,[1] Raj Tummala,[1] Johann E. Gudjonsson,[2] Charles Ross,[2] Gwendolyn Binder,[1] David Chang,[1] Samik Basu,[1,4] and Dinesh Khanna[2,4]

[1]Cabaletta Bio, Philadelphia, PA, USA; [2]University of Michigan, Ann Arbor, MI, USA

Under compassionate use, CAR T cells have elicited durable remissions in patients with refractory systemic sclerosis (SSc). Here, we report on the safety, efficacy, and correlative data of the first SSc subject treated with a fully human CD19-CART cell therapy (resecabtagene autoleucel) in the RESET-SSc trial (NCT06328777). Nine days post-infusion, the subject experienced a brief grade 2 episode of cytokine release syndrome that resolved promptly with intravenous hydration and indomethacin. Immune-effector-cell-associated neurotoxicity syndrome was not observed. Seven days post-infusion, the subject experienced a brief grade 4 episode of neutropenia that resolved rapidly with a single dose of colony-stimulating factor. Despite discontinuation of all immunosuppressants since infusion, over the 9-month period post-infusion, the subject's skin thickness decreased, pulmonary function improved, and nailfold vasculature improvement was observed. Following infusion, B cells were depleted both in the periphery and within secondary lymphoid tissue. B cell aplasia was observed by day 13 post-infusion. Furthermore, serum BAFF was highly elevated. B cell repopulation began 8 weeks post-infusion, with cells exhibiting a transitional naive phenotype. Autoantibodies to RNA polymerase III decreased to undetectable levels. The infusion product consisted of predominantly CD4^{+} T cells, and both CD4^{+} and CD8^{+} populations had similar *in vitro* activity. Post-infusion, rese-cel expansion peaked at 13 days. These data detail the safety, efficacy, and pharmacodynamics of rese-cel in the first SSc subject.

INTRODUCTION

Systemic sclerosis (SSc) is a rare, heterogeneous, and often fatal multisystem chronic autoimmune disorder with a complex pathogenesis. It has the highest mortality of any rheumatic disease.[1] Abnormal B cell function contributes to the disease pathology, and disease-specific autoantibodies, such as anti-centromere, anti-topoisomerase I (anti-Scl70), and anti-ribonucleic acid (RNA) polymerase III, are often present.[2] Disease features include progressive skin fibrosis, viscera and organ fibrosis, and diffuse fibroproliferative vasculopathy, which often manifest as thickened skin, interstitial lung disease, pulmonary hypertension, decreased gastrointestinal motility, Raynaud's phenomenon, and digital ischemic events, among others.[3] SSc is subdivided into diffuse cutaneous (dcSSc) and limited cutaneous (lcSSc) subtypes, where dcSSc has more extensive skin fibrosis to the trunk and proximal extremities, in addition to the fingers, distal extremities, and face. Patients with dcSSc also tend to have more rapid disease progression and early development of visceral organ complications compared to lcSSc.[4]

Treatments for SSc aim to control disease activity, limit progression of organ damage, and decrease morbidity and mortality. However, many fail to provide durable clinical responses without chronic immunosuppression. A recent analysis of an observational prospective multicenter cohort of patients with dcSSc demonstrated very little clinical improvement in the skin and lung pathology, with ongoing clinical progression of SSc, as well as a high mortality rate despite immunosuppressive use in this population.[5] Dysregulation of both innate and adaptive immunity is paramount to SSc pathogenesis, with B cells playing a predominant role in disease manifestations.[2,6,7] In addition to associations of specific autoantibodies with disease subtypes, susceptibility to the development of SSc has been associated with multiple B-cell-associated proteins. Abnormal B

Received 26 September 2025; accepted 18 December 2025;
https://doi.org/10.1016/j.omta.2025.201663.

[3]These authors contributed equally

[4]Senior author

Correspondence: Samik Basu, Cabaletta Bio, Philadelphia, PA, USA.
E-mail: samik.basu@cabalettabio.com
Correspondence: Dinesh Khanna, University of Michigan, Ann Arbor, MI, USA.
E-mail: khannad@med.umich.edu

 © 2025 The Author(s).
Published by Elsevier Inc. on behalf of The American Society of Gene and Cell Therapy.
This is an open access article under the CC BY license (http://creativecommons.org/licenses/by/4.0/).

cell function, other than autoantibody production, also plays an important role in the multifactorial etiology of SSc.[8,9]

Given the role of B cells in SSc pathogenesis, B cell depletion strategies have been evaluated. Clinical trials in SSc with the B cell depleting antibody rituximab have shown some effectiveness.[10,11] A systematic review and meta-analysis consisting of 20 studies involving 575 rituximab-treated SSc patients who had lung involvement showed improved forced vital capacity (FVC) and diffusion capacity of the lungs for carbon monoxide (DLCO) following rituximab treatment. However, the two randomized controlled trials included in this meta-analysis showed no difference in FVC and DLCO in the rituximab-treated and control groups at 12 months post-treatment.[12] Despite therapeutic impact in some patients, rituximab therapy primarily remains non-curative, potentially due to limited antibody penetrance into secondary lymphoid compartments.[13] Inadequate response is associated with incomplete disruption of the B cell repertoire, suggesting maintenance of autoreactive clones.[14]

Chimeric antigen receptor (CAR) T cells targeting CD19 have been shown to eliminate B cells both in the peripheral blood and secondary lymphoid organs.[15] Furthermore, CD19-targeting chimeric antigen receptor (CAR) T cells produced durable, drug-free responses in SSc patients in academic studies.[16,17] Resecabtagene autoleucel (rese-cel) is a fully human, autologous CD19-directed 4-1BBζ CAR T cell therapy, designed to deeply and transiently deplete $CD19^+$ B cells.[18,19] RESET-SSc (NCT06328777) is an ongoing phase I/II trial evaluating rese-cel in two cohorts of adults with SSc and either severe skin (SSc-Skin) or organ (SSc-Organ) involvement. Here, we provide the first report on the emerging clinical and translational data from the first patient dosed in the RESET-SSc clinical trial.

RESULTS

Clinical course and efficacy outcomes following rese-cel infusion in a subject with SSc

A 66-year-old female presented with a 2-year history of diffuse cutaneous SSc. She was diagnosed in April 2022 with Raynaud's phenomenon, progressive skin thickening, positive ANA (1:1,280 by indirect immunofluorescence on HEP-2000 cells with a speckled pattern) and positive anti-RNA polymerase III antibodies. Prior to enrollment, the subject was treated with mycophenolate mofetil 1,500 mg twice daily (started April 2022), hydroxychloroquine 300 mg (started February 2024), and investigational brentuximab vedotin as part of a clinical trial (initiated September 2022 and last infusion February 2023). She continued to have worsening of her skin disease (as assessed by the modified Rodnan skin score [mRSS]), a clinical assessment of skin thickness,[20] and functional disability (as assessed by the health assessment questionnaire-disability index [HAQ-DI][21,22]) (Figure S1). The subject also had small and large joint contractures, inflammatory arthritis, and interstitial lung disease. Due to ongoing worsening skin, HAQ-DI, and overall well-being despite SSc-specific therapies, the patient was enrolled in the RESET-SSc trial. Following discontinuation of mycophenolate mofetil and standard preconditioning regimen with fludarabine (25 mg/m^2 daily for 3 days) and cyclophosphamide (1,000 mg/m^2 once) on days −5 through −3, the patient was infused with 1×10^6 CAR T cells per kilogram body weight (a total dose of 9.18×10^7 cells). A transient grade 4 neutropenia was observed 7 days after infusion, which resolved with a single dose of growth colony-stimulating factor (Figure 1A). The patient experienced grade 2 cytokine release syndrome (CRS) manifesting as fever and transient hypotension 9 days following rese-cel infusion. The hypotension resolved promptly following intravenous (i.v.) hydration, and the fever resolved following the use of indomethacin, without the use of tocilizumab, 2 days later without sequelae (Figure 1A). The patient did not experience immune-effector-cell-associated neurotoxicity syndrome (ICANS), any serious infection, or other serious treatment-related adverse events. The patient showed marked improvement on the mRSS, from the pre-infusion time point through 36 weeks post-infusion, with improvement in the score from 42 to 28 points throughout the follow-up time (Figures 1B and 1C). The patient also exhibited modest improvement in pulmonary function as measured by percent predicted FVC and DLCO as early as 12 weeks post-infusion, which was sustained through the first 24 weeks post-infusion (Figure 1H) with stabilization of her underlying interstitial lung disease on computed tomography (CT) of the chest. Moreover, the patient achieved a sustained, drug-free revised Composite Response Index in Systemic Sclerosis (rCRISS-25)[23] response starting at week 12. Furthermore, there were improvements in other key clinical assessments, including the HAQ-DI, the Physician and Patient Global Assessment (PGA and PtGA), Pain, and Functional Assessment of Chronic Illness Therapy-Fatigue (FACIT-F) scores (Figures 1D–1G). Moreover, indirect evidence of vascular recovery or stabilization via nailfold capillaroscopy (NFC) was observed at 24 weeks post-infusion. Specifically, the number of capillaries with abnormal morphology or enlarged dimensions decreased per 1,000 μm field. Furthermore, the capillary density per 1,000 μm field increased following rese-cel infusion (Figure 1I).

Rese-cel drug product characterization and post-infusion pharmacokinetics and pharmacodynamics

The rese-cel infusion product (IP) consisted of 65.0% CAR^+ T cells that were 90.6% $CD4^+$ and 8.9% $CD8^+$; 89.6% of the CAR^+ T cells were of the central memory phenotype ($CCR7^+CD45RA^-CD95^-$), and 68.6% of the cells were $HLA\text{-}DR^+$ (Figures 2A, S2A, and S2B). The corresponding apheresis material consisted of predominantly naive phenotype ($CD45RA^+CCR7^+CD95^-$) $CD4^+$ T cells that expressed low levels of HLA-DR (Figures 2A, S2A, and S2B). Both the $CD4^+$ and $CD8^+$ fractions of rese-cel exhibited cytolytic activity *in vitro* (Figure 2B). Following infusion, CAR^+ T cells were detected by digital polymerase chain reaction (dPCR) in the circulation of the subject at days 6, 7, 13, and 21 post-infusion (Figure 2C). Circulating CAR T cell concentration peaked at 76 cells per microliter of blood (Cmax) on day 13 (Figure 2C). At peak expansion, CAR T cells comprised 4.4% of the total T cells in circulation as determined by flow cytometry (Figure 2A). In contrast to the rese-cel IP, circulating CAR T cells at day 13 were predominantly effector memory ($CCR7^-CD45RA^-$) $CD8^+$ T cells (Figure 2A); 96.3% of the

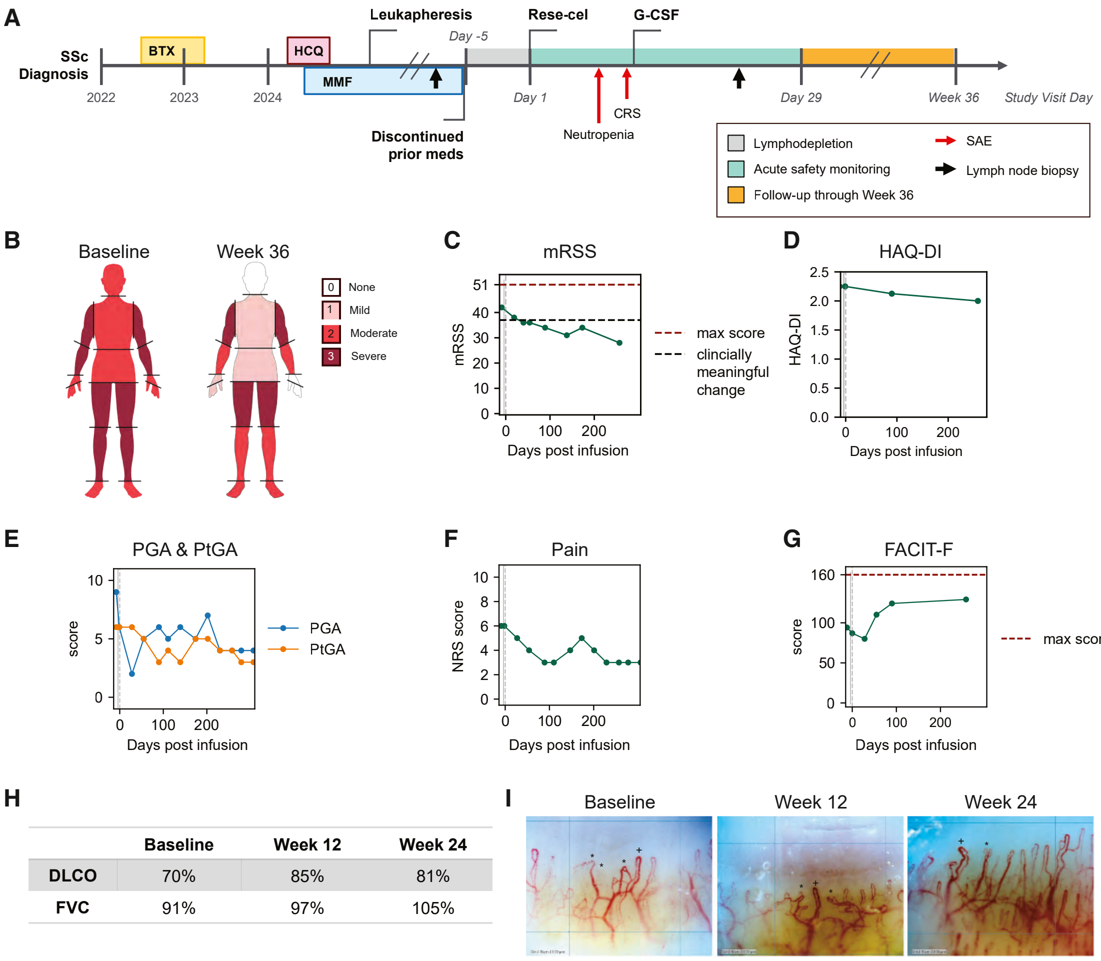

	Baseline	Week 12	Week 24
DLCO	70%	85%	81%
FVC	91%	97%	105%

Figure 1. Clinical course and efficacy outcomes following rese-cel infusion in subject with SSc

(A) Schematic representation of the SSc patient's clinical course prior to and following rese-cel. The patient was diagnosed 2 years prior to rese-cel infusion and was refractory to brentuximab vedotin (BTX; yellow box), hydroxychloroquine (HCQ; pink box), and mycophenolate mofetil (MMF; blue box). Cessation of all medications before infusion. The SSc subject underwent 3 days of preconditioning lymphodepletion (gray shading) prior to rese-cel infusion on study visit day 1. The subject experienced two serious adverse events (represented by red arrows) within the first 28 days following infusion (represented by green shading). Neutropenia was observed 7 days post-infusion, which resolved after a single dose of granulocyte colony-stimulating factor (G-CSF), and CRS (grade 2) was observed 9 days post-infusion. Lymph node biopsies were obtained prior to lymphodepletion (baseline) and 21 days following rese-cel infusion (represented by black arrows). Per study protocol, the subject participated in additional monitoring visits through week 36 post-infusion. (B) Visual representation of the modified Rodnan skin score (mRSS) by body area at baseline and 36 weeks after rese-cel infusion. (C) mRSS scoring over time from baseline through 36 weeks post-infusion. (D) Health Assessment Questionnaire-Disability Index (HAQ-DI) scoring over time from baseline through 36 weeks post-infusion. (E) Physician and Patient Global Assessment (PGA and PtGA) scoring from baseline through 36 weeks post-infusion. (F) Pain as measured on a Numeric Rating Scale (NRS) from baseline through 36 weeks post-infusion. (G) Functional Assessment of Chronic Illness Therapy (FACIT-F) scoring from baseline through 36 weeks post-infusion. (H) Pulmonary function tests, percent predicted diffusing capacity of the lungs for carbon monoxide (DLCO), and percent predicted forced vital capacity (FVC) at baseline levels, 12 weeks post-infusion, and 24 weeks post-infusion. (I) Nailfold capillary images of the 4th left digit, used to assess vasculature, at baseline (left panel), 12 weeks post-infusion (middle panel), and 24 weeks post-infusion (right panel). Symbols: * designates abnormal capillary morphology, and + designates enlarged capillary dimension. Each grid represents 1,000 μm.

circulating CAR T cells expressed HLA-DR, suggesting cellular activation (Figure 2A). Serum interferon gamma (IFN-γ) peaked on day 8 (523.5pg/mL) before CAR T cell peak expansion in the peripheral blood (Figure 2D) while serum levels of IP-10 (CXCL10) and IL-12p40 peaked on day 13, concurrent with C_{max} (Figure 2D). Serum interleukin-6 (IL-6) peaked on day 13 post-infusion as well, whereas serum IL-8 peaked earlier on day 8 (Figure 2D). Serum levels of pro-inflammatory cytokines stabilized after the first month post-infusion (Figure S3).

Impact of rese-cel on the B cell compartment and associated serologic changes

Following rese-cel infusion, circulating B cells decreased rapidly and were undetectable by day 13 (Figure 3A) and remained undetectable

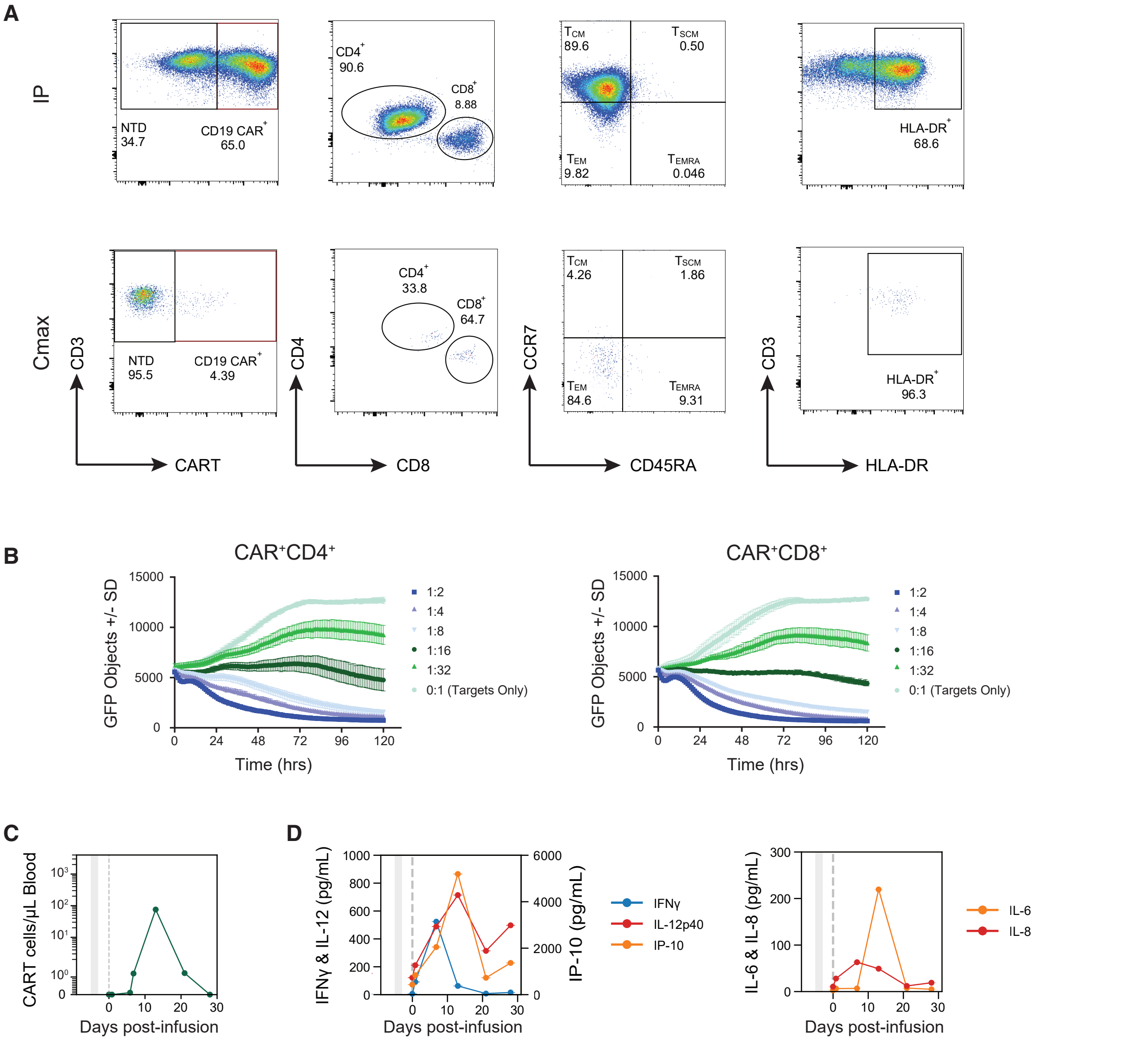

Figure 2. Rese-cel drug product characterization and post-infusion pharmacokinetics and pharmacodynamics

(A) Flow cytometric characterization of Rese-cel in infusion product (IP) (top panels) and in circulation at C_{max} (bottom panels). CD4, CD8, CCR7, CD45RA, and HLA-DR expression within CD3-positive and CAR-positive cells is shown. Left panels: CD3 (*y* axis) versus CAR T (*x* axis); second from left panels: CD4 (*y* axis) versus CD8 (*x* axis); third from left panels: CCR7 (*y* axis) versus CD45RA (*x* axis); right panels: CD3 (*y* axis) versus HLA-DR (*x* axis). For third panels from left, populations represented as follows: T_{EM} ($CD45RA^{-}CCR7^{-}$), T_{EMRA} ($CD45RA^{+}CCR7^{-}$), T_{CM} ($CD45RA^{-}CCR7^{+}$), and T_{SCM} ($CD45RA^{+}CCR7^{+}$). (B) Rese-cel infusion product lysis of $CD19^{+}GFP^{+}$ target Nalm6 cells. Rese-cel infusion product in (A) was sorted into CD4 (left panel) and CD8 fractions (right panel). Cell lysis curves represented by number of GFP^{+} target cells over 120 h at effector-to-target ratios ranging from 0:1 to 0.5:1. (C) Pharmacokinetic response of rese-cel in SSc subject represented as number of CAR T cells/μL blood from baseline through 29 days post-infusion. (D) Serum concentrations of IFN-γ, IP-10 (CXCL10), and IL-12p40 before and after rese-cel infusion (left panel). Serum concentrations of IL-6 and IL-8 before and after rese-cel infusion (right panel).

through day 28 (Figure 3B). B cell reduction was accompanied by a commensurate increase in serum B cell activating factor (BAFF) levels (~18-fold higher than baseline) within the first month after rese-cel infusion (Figure 3B). B cells began repopulating peripherally by week 8 (Figure 3B), whereas leukocyte counts normalized post-preconditioning by day 13 following rese-cel infusion (Figure 3C). Similar to the inverse relationship between B cells counts and BAFF levels, serum IL-15 concentrations increased during peripheral leukocyte reduction and normalized with cellular repopulation (Figure 3C). Consistent with peripheral B cell aplasia, B cells were undetectable in an inguinal lymph node biopsy collected on day 21 determined via immunohistochemistry (Figure 3D). Spatial sequencing of the biopsy showed a >99% reduction in the number of cells (from ~5,000 cells pre-infusion to ~20 cells post-infusion)

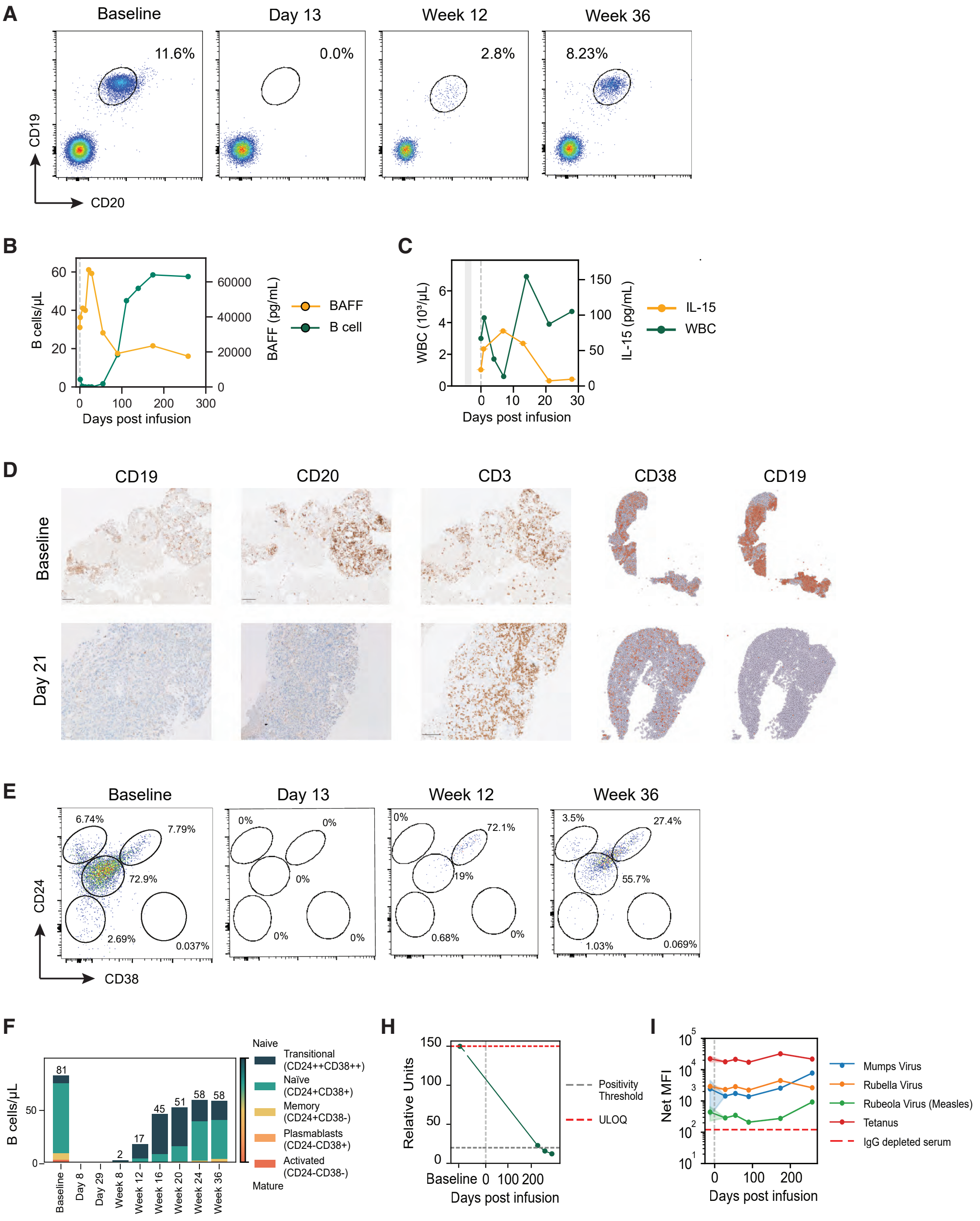

Figure 3. Impact of rese-cel on the B cell compartment and associated serologic changes

(A) B cells identified by flow cytometry at baseline through week 36 after rese-cel infusion in peripheral blood. B cells show in the oval gate as the percentage of live, single lymphocytes expressing CD19 (*y* axis) and CD20 (*x* axis). (B) B cell counts and serum BAFF levels from baseline though 36 weeks post-infusion overlaid as line plots. Left *y* axis represents serum BAFF levels in pg/mL (yellow) and right *y* axis represents B cell counts as number of CD19^{+} CD20^{+} cells/μL blood (green). (C) Leukocyte counts and serum IL-15 levels overlaid as line plots overtime. Left *y* axis represents leukocyte counts as thousand cells/μL blood (green) and right *y* axis represents IL-15 levels in pg/mL (yellow). The *x* axis represents time in days from baseline with vertical gray shaded lines indicating lymphodepletion and vertical gray dotted lines representing rese-cel infusion in both B and C. (D) B cells and T cells identified by immunohistochemistry analysis from inguinal lymph node biopsy at baseline (top panels) and 21 days post-infusion (bottom panels). B cells characterized via CD19 (left panels) and CD20 (2nd panels from left). T cell characterized via CD3 (center panels). B cells and plasma cells identified by spatial

(legend continued on next page)

containing CD19 mRNA transcripts following rese-cel infusion (Figure 3D). Notably, the number of $CD3^+$ T cells did not significantly change in the tissue (Figure 3D). Spatial sequencing of the biopsy showed a mild, ∼22%, reduction in the number of cells containing CD38 mRNA transcripts (from ∼900 cells pre-infusion to ∼700 cells post-infusion) after rese-cel infusion (Figure 3D). Repopulating B cell phenotypes were mostly transitional naive through week 20 (Figures 3E and 3F). By week 24, a majority of the circulating B cells were mature naive ($CD24^+CD38^+$) (Figures 3E and 3F). Following infusion, anti-RNA polymerase III antibody levels decrease dramatically from >150 units at baseline to below the lower limit of detection (<20 units by ELISA) by 36 weeks post-infusion (Figure 3H). In contrast, serum levels of vaccine-associated antibodies remained stable over the same post-infusion time period (Figure 3I).

DISCUSSION

This report of CD19-CAR T treatment in an SSc subject contributes to the body of literature showing the tolerability and efficacy of CAR T cells for treating systemic sclerosis.[16,24,25] The data presented here provide greater insight into the mechanism of action of 4-1BBζ CD19-CAR T cells in SSc. To our knowledge, this is the first published report in an SSc subject in the setting of a phase I/II clinical trial (NCT06328777), in contrast to previous reports from compassionate use protocols.[16,24,25] Patients with dcSSc have limited treatment options and face a high mortality rate due to progressive organ fibrosis and dysfunction. Current treatment options are primarily limited to immunosuppressants and targeted biologic agents, which are frequently used off-label, require chronic administration, and only minimize disease impact and development of major complications.[26] These therapies offer only modest benefit in delaying disease progression, and the prognosis for patients with progressive SSc disease remains poor.[27,28] The subject described in this report had SSc refractory to several lines of therapies including immunomodulatory medications, prior to their infusion with rese-cel.

Despite discontinuation of all immunosuppressants, we observed a rapid and deep B cell depletion following and rese-cel administration that occurs simultaneously with rese-cel expansion (Figures 2A, 2C, 3A, 3B, and 3D). The patient developed grade 2 CRS manifesting as fever and transient hypotension, which was responsive to i.v. fluids and indomethacin but did not require tocilizumab. The depth of B cell depletion observed in the periphery is supported by the contemporaneous absence of B cells in the lymph node (Figures 3A, 3B, and 3D). Moreover, the B cell depletion was accompanied by a substantial increase in serum BAFF levels (Figure 3B), which has been observed as a homeostatic response to B cell depletion.[18,29] BAFF is produced by a wide variety of leukocyte populations and supports B cell and plasma cell homeostasis and proliferation.[30] Although increases in serum BAFF have been observed in autoimmune patients treated with B cell depleting antibodies, no publications to date have reported levels as high as we have observed in our first SSc subject. Indeed, most reports show an increase in serum BAFF from 2- to 8-fold following the use of B cell depleting antibodies, with peak concentrations ranging from 5,000 to 14,000 pg/mL,[31–34] whereas we observed a serum BAFF induction of ∼18-fold with a peak concentration of nearly 70,000 pg/mL (Figure 3B). Markedly elevated serum BAFF may be a useful biomarker of secondary lymphoid tissue B cell depletion. Consistent with a standard preconditioning regimen used, leukocyte levels reached a nadir 7 days following infusion and recovered within 13 days of rese-cel infusion (Figure 3C). Given that B cells remained undetectable from day 13 through week 8, these data support the selective elimination of B cells by rese-cel in this subject. The first detectable peripheral blood B cells to emerge following depletion are recent bone marrow emigrants phenotypically defined as transitional naive B cells.[35] These results are consistent with prior reports of B cell repopulation in subjects post-CAR T cell infusion.[18] Moreover, our results mirror those seen in patients who receive an allogenic hematopoietic stem cell transplant (aHSCT) where all pre-existing B cells are chemotherapeutically destroyed and the patient is fully reconstituted with new B cells originating from bone marrow progenitors.[36] Together, the level of BAFF induction, peripheral B cell counts, and lymph node B cell evaluation provide strong evidence of rese-cel's ability to deplete B cells throughout the body and improved penetrance to tissues as compared to antibody- or protein-based therapeutics.

Following infusion, we observed a decrease in anti-RNA-Pol III antibodies without a decrease in vaccine or infectious-disease-associated antibodies, consistent with other reports of autoimmune disease patients treated with CD19-CAR T cells (Figures 3H and 3I).[18,29] The reduction of anti-RNA-Pol III antibodies from >150 U (strong positive) to <20 U (normal healthy donor range) suggests that the autoantibody-secreting cells are either $CD19^+$ or short-lived $CD19^-$ plasma cells, whereas the vaccine or infectious-disease-antibody-secreting cells likely arise from $CD19^-CD38^+$ long-lived plasma cells residing in secondary lymphoid tissue or bone marrow niches.[37] In this report, we add to the evidence in autoimmune disease that CD19-directed CAR T cells do not deplete reservoirs of long-lived plasma as $CD38^+$ cells are not impacted by rese-cel (Figure 3G). In addition to a decrease in autoantibodies, a decrease in skin thickness as determined by the mRSS score and clinical improvement in pulmonary function were observed, which

RNA-sequencing analysis from inguinal lymph node biopsy at baseline and 21 days post-infusion. B cells characterized via CD19 (right panels) and plasma cells characterized via CD38 (4th panels from left). (E) Maturation status of B cells as determined by flow cytometry at baseline though 36 weeks post-infusion determined by CD24 and CD38 expression. Subpopulation as follows: (I) memory B cells, (II) transitional naive (T1 and T2) B cells, (III) activated naive B cells, (IV) activated naive or activated memory B cells, (V) plasmablasts or pre-plasmablasts. (F) Stacked bar plot depicting B cell maturation phenotype determined by CD24 and CD38 expression and total $CD19^+$ $CD20^+$ B cell counts in cells/μL of blood, depicted numerically above each bar. (H) Serum levels of anti-RNA polymerase III antibodies at baseline through 36 weeks after rese-cel infusion. Antibodies measured via ELISA. (I) Serum levels of vaccine-associated autoantibodies at baseline through 36 weeks after rese-cel infusion. Antibodies measured by Luminex and represented as net median fluorescence intensity..

contributed to achievement of a sustained, drug-free revised CRISS-25 response starting 12 weeks following infusion and continuing through week 24 thus far (Figures 1B, 1C, and 1H). The revised CRISS-25 is a validated composite endpoint being used in registrational trials of SSc, whereby responders must achieve response in at least two core set measures and worsening in no more than one core measure (with improvement of worsening based on a change of at least 25% in mRSS, HAQ-DI, Patient Global Assessment, Physician Global Assessment, or at least 5% in percent predicated FVC).[23,38] Furthermore, patients cannot develop significant SSc events such as new renal crisis or worsening organ function. Notably, we also observed indirect evidence of vascular stabilization via NFC (Figure 1I). Lastly, given the use of pre-conditioning prior to rese-cel infusion, it is possible that fludarabine and cyclophosphamide may have partially contributed to the aforementioned reduction in auto-antibody levels and clinical improvements.

Serum IFN-γ peaked before rese-cel was detectable in circulation. IFN-γ is released by CAR T cells upon target engagement (Figure 2D), suggesting the possibility that CAR T cells engaged their target $CD19^+$ B cells in the peripheral blood and lymphoid tissue within the first 8 days after infusion prior to being present in circulation at high enough levels for detection. The delayed increases in serum IL-6, IP-10, and IL-12p40 that peak in parallel CAR T cell C_{max} on day 13 support prior CAR T cell activation and proliferation (Figures 2D and 2E). These cytokines are likely secreted from myeloid cell populations in response to CAR-T-cell-secreted IFN-γ. The peaks in IFN-γ and IL-6 are likely responsible for the subject's episode grade 2 CRS, which was contemporaneous with these elevations. Elevations in IFN-γ, IL-6, and IL-8 have been associated with CRS in oncology patients. Importantly, the peak serum concentration of IL-6 in this patient is much lower than the concentrations in oncology patients who experience CRS following CD19 CAR T cell treatment.[39] The infusion product was composed of predominantly $CD4^+$ CAR^+ T cells (Figure 2A), reflecting the $CD4^+$ T cell dominant nature of this subject's apheresis product (Figure S2). In autoimmune patients, infusion products tend to be $CD4^+$ T-cell dominant. This observation is largely attributed to a higher CD4:CD8 ratio in the starting leukapheresis.[18,40] The CAR^+ T cells in circulation were mostly $CD8^+$ and exhibited an effector memory ($CCR7^-$) phenotype potentially suggesting a preferential expansion of $CD8^+$ CAR T cells or preferential trafficking of these cells to the periphery (Figure 2A). It is possible that $CD4^+$ CAR T cells preferentially traffic to secondary lymphoid tissues due to higher expression of CCR7 and lyse target B cells, given the comparable lytic capacity of $CD4^+$ and $CD8^+$ CAR T cells *in vitro* (Figures 2A and 2B), despite only being detected at low levels in circulation.

In summary, our data provide mechanistic insights into the activity of rese-cel, a fully human CD19 4-1BBζ CAR T, in SSc. We show that rese-cel was well tolerated, can rapidly eliminate target $CD19^+$ B cells in both the periphery and secondary lymphoid tissues post-infusion, reduce disease-associated autoantibodies without impacting pre-existing humoral immunity, and can lead to meaningful skin improvement in SSc patients despite discontinuation of all immunosuppressants. Furthermore, we have identified a novel approach to further define the pharmacodynamics of rese-cel infusion by assessing for marked elevations in serum BAFF in combination with depletion of peripheral B cells and elevations in serum IFN-γ prior to expansion of T cells in the periphery. These findings demonstrate the potential of rese-cel for SSc patients who are refractory to therapies. Based on these encouraging data in the context of a Phase I/II trial, treating additional SSc patients is warranted.

MATERIALS AND METHODS

Study design and participants

Eligible subjects had a clinical diagnosis of SSc based on the 2013 American College of Rheumatology/European League Against Rheumatism classification criteria. Furthermore, subjects had to have evidence of moderate to severe active skin or organ disease, despite prior or current treatment with standard-of-care treatments.

The clinical trial (NCT06328777) is a phase I/II, multi-center, open-label study. The primary objective is to evaluate the safety and tolerability of rese-cel in subjects with active SSc over 28 days post-infusion (study visit day 29, per protocol). Selected secondary objectives include, but are not limited to, evaluating the effects of rese-cel on peripheral B cell counts, peripheral leukocyte counts, SSc serology, and SSc disease activity over 156 weeks post-infusion (up to 36 weeks is included herein). mRSS assessments were determined as previously described.[20] HAQ-DI, PGA, PtGA, Pain, and FACIT scores were determined as previously described.[21,22,41–43] The FACIT and all related works are owned and copyrighted by, and constitute the intellectual property of David Cella, Ph.D. Permission for use of the FACIT-F questionnaire is obtained by contacting Dr. Cella at information@facit.org. English (Universal) Copyright 1987, 1997 by David Cella, Ph.D. The Relevant secondary and exploratory objectives include evaluating the presence and phenotype of rese-cel cells post-infusion and the activity of rese-cel cells post-infusion.

Briefly, following the identification and consent of eligible subjects, subjects undergo leukapheresis prior to infusion for collection of peripheral blood mononuclear cells (PBMCs). Following rese-cel manufacture and infusion, subjects were followed for 28 days following rese-cel infusion for safety and scheduled research assessments. Serum and PBMCs were collected at baseline (prior to lymphodepletion), pre-infusion (after lymphodepletion), and at 1, 4, 7, 14, 21, and 28 days, and 8, 12, and 16 weeks after rese-cel infusion. Clinical assessments were conducted at baseline (prior to lymphodepletion), 12, 24, and 36 weeks after rese-cel infusion.

Lymph node biopsy

Ultrasound examination of the inguinal region was performed to identify the largest lymph node. In the Interventional Radiology suite at Michigan Medicine, the patient was placed in the supine position, and local anesthesia was administered to the inguinal area. Under sterile conditions, 3–4 core tissue samples were obtained from the targeted lymph node using an 18-gauge core biopsy needle. The

patient was subsequently monitored for 1 h post-procedure. The biopsies collected were immediately transferred into HypoThermosol, and then stored at 4°C for transportation to research laboratories, and processed within 30 min of collection.

Nailfold capillaroscopy

Nailfold capillaroscopy (NFC) is a rapid and easily applicable tool that allows direct visualization of nailfold capillaries.[44] Nailfold capillary vessels run parallel to the skin surface, allowing for direct vascular visualization. In SSc and sclerodermatous diseases, there are identified patterns that occur on NFC, allowing further characterization of vascular involvement.[45] This allows SSc-specific vascular evaluation in a standardized fashion. We applied 200× magnification to nailfold capillaries via Inspectis CapiScope equipment. Digits 2 through 5 of the bilateral hands were examined, and at least two images from each nail bed were obtained. Baseline NFC images were acquired prior to infusion, with repeat NFC imaging performed at 3 and 6 months post-rese-cel infusion. NFC in this patient revealed indirect evidence of vascular stabilization.

Rese-cel manufacturing and dosing

The rese-cel product was manufactured using a previously reported protocol.[46] Briefly, T cells were selected/activated using anti-CD3/CD28 microbeads (Gibco) at a bead:cell ratio of 3:1. Lentiviral vector that carries target CAR was added within 24 h after activation, and T cells were expanded in the Xuri bioreactor (Cytiva). The harvested cells were resuspended in the final formulation reagent CryoStor B/CryoStor 10 (BioLife Solutions) and then formulated into cryobags, as drug product, as well as into vials for correlative tests. The drug product was then frozen to −90°C using a controlled rated freezer (CRF) and stored at ≤ −130°C. Patients received a preconditioning regimen consisting of fludarabine (25 mg/m^2 on days −5, −4, and −3) and cyclophosphamide (1,000 mg/m^2 on day −3) prior to a single infusion of rese-cel at 1 × 10^6 cells/kg.

Flow cytometry

PBMCs were labeled with anti-human antibodies and reagents to measure B cells and CAR T cell phenotype. B cell characterization was performed using anti-CD19 (PE-Fire 700; BioLegend, clone: HIB19, RRID: AB_2876589), anti-CD20 (Brilliant Violet 570; BioLegend, clone: 2H7, RRID: AB_2563805), Fixable Viability Dye eFluor 506 (Thermo Fisher Scientific), anti-CD24 (APC-Fire 750; BioLegend, clone: ML5, RRID: AB_2750463), anti-CD38 (Brilliant Violet 711; BioLegend, clone: HIT2, RRID: AB_2563811), anti-IgG (Brilliant Violet 421; BioLegend, clone: M1310G05, RRID: AB_2565626), and anti-IgD (PE-Cyanine 7; BioLegend, clone: IA6-2, RRID:AB_10680462). CART cell detection was performed with anti-CD3 (APC-Vio770; BioLegend, clone: SK7, RRID: AB_2725964), anti-CD4 (PerCP-Vio700; Miltenyi, clone: SK3, RRID: AB_2726039), anti-CD8 (APC; Miltenyi, clone: HIT8a, RRID:AB_2659237), anti-CCR7 (PE-Vio770; Miltenyi, clone: G043H7, RRID: AB_2659237), anti-CD45RA (VioBlue; Miltenyi, clone: T6D11, RRID: AB_2889586), anti-HLA-DR (PE-Vio615; Miltenyi, clone: L243, RRID: AB_2652166), Fixable Viability Dye eFluor 506 (Thermo Fisher), and CD19-CAR detection reagent (Miltenyi Biotech). PBMCs were incubated with fluorescently labeled antibodies for 30 min at 4°C in the dark. Samples were washed and incubated with secondary antibody (anti-Biotin PE; Miltenyi, clone: Bio3-18E7, RRID: AB_2661378) for 30 min. Labeled samples were acquired on the NovoCyte Quanteon flow cytometer (Agilent) and analyzed using FlowJo software (FlowJo). The absolute B cell count per microliter (B cells/μL) was calculated by multiplying the percentage of CD19$^+$/CD20$^+$ B cells determined by flow cytometry by absolute lymphocyte count in thousands of cells/μL obtained from complete blood count (CBC) and scaling by a factor of 1,000 divided by 100.

Serum cytokine assessment

Serum cytokine levels were quantified using Meso Scale Discovery (MSD) electrochemiluminescence multiplex platform. Serum samples were tested in triplicate using the VPLEX pro-inflammatory panel, cytokine panel 1, chemokine panel 1, and a custom UPLEX measuring B cell activating factor (BAFF). Serum samples were tested following the optimization of the manufacture's recommended protocols at 1:4 and 1:100 dilutions for the VPLEX and UPLEX assays, respectively. Serum cytokine concentrations are reported in pg/mL.

Leukocyte counts

Leukocyte counts were measured by clinical complete blood count (CBC) test run locally at the clinical site.

Serum antibody assessment

Serum vaccine and pathogen-associated antibody titers were quantified using Luminex FlexMap technology (Luminex Corporation). A custom Luminex assay was used to measure various antibodies to vaccines.[29] Serum samples were incubated in duplicate with antigen-bound beads and analyzed using Luminex FlexMap instrument. Antibody reactivity values are represented as median fluorescence intensity (MFI). Serum RNA polymerase III antibody titers were quantified using ELISA (Inova Diagnostics). Briefly, serum samples were incubated for 30 min at room temperature on pre-coated RNA-polymerase-III-coated plates, washed, and then incubated again for 30 min with horseradish-peroxidase-labeled goat anti-human immunoglobulin G (IgG) (secondary antibody). Samples were then washed again and incubated with TMB chromogen for 30 min at room temperature prior to addition of stop solution. Samples were analyzed via plate reader. Reference values are as follows: <20.0 U (negative), 20.0–39.9 U (weak positive), 40.0–80.0 U (moderate positive), and >80.0 U (strong positive).

Cytolytic function of drug product

Cryopreserved drug product was thawed, washed, and rested at 37°C overnight. CD19-CAR T cells were sorted into CD4$^+$ and CD8$^+$ fractions on the Tyto cell sorter (Miltenyi) prior to co-culture, with clonal Nalm6 cells expressing CD19 and green fluorescent protein (GFP) at various effector (CAR T cell) to target cell ratios in triplicate (0.5:1, 0.25:1, 0.125:1, 0.0625:1, 0.0312:1, and 0:1 or Target only).

Mixed cell populations were incubated for 5 days at 37°C in the Incucyte live cell imaging platform (Sartorius) with five images per well taken every hour. The number of GFP$^+$ target cells were enumerated from images and reported as averages over the 120-h incubation.

Digital PCR

Peripheral CAR T cells were quantified using digital PCR (dPCR). DNA was extracted from PBMCs using the QIAamp DNA Blood Mini Kit (Qiagen) following the manufacturer's protocol. Following concentration measurement on the Nanodrop One (Thermo Fisher Scientific), samples were diluted to 30 ng/μL in TE buffer. Nanoplate-based dPCR was performed on a QIAcuity Four instrument (Qiagen) in a duplex assay measuring IC78 (CAR sequence; F: ACCAAGGTCACCGTCCTA; R: GCTGTATCCAGAACCCTTACAG; P: TTTCACCTCTGCTCCAGACTGCAC) and RPP30 (endogenous control; F: CCAAGAAAGCCAAGTGTGAG; R: TTTGTTGTGGCTGATGAACTAT; P: TGTCAGCACCCTTCTTCCCTTT). One hundred fifty nanograms of DNA were analyzed per reaction; samples were analyzed in triplicate. dPCR thermal cycling parameters were 2 min at 95°C, 40 cycles of 95°C for 15 s, and 62°C for 30 s. Results less than 1 copy/μL were reported if found to be statistically different from the background signal of the assay.[47] Copy number of IC78 per μg of DNA was calculated for each sample, normalized to RPP30.[48] CAR T cell concentrations (cells/μL blood) were calculated using patient's peripheral blood mononuclear cell (PBMC) count and the vector copy number (VCN) measured in the infusion product. The calculation was performed as follows: CAR T cells/μL blood = (CAR copies/μg DNA) × (1 μg DNA/1×10^5 cells) × (PBMC/μL blood) × (1/VCN). An estimate of 1 μg DNA per 1×10^5 cells was used, and PBMC counts were approximated by summing lymphocyte and monocyte counts.

Immunohistochemistry

Lymph node biopsies were fixed in formalin for 3 h at room temperature, then transferred to 70% ethanol and stored at 4°C. Within 3 days, samples were embedded into FFPE blocks by the University of Michigan Orthopedic Research Laboratories Histology Core and stored at 4°C. Sections (5 μm thick) were prepared. Immunohistochemistry was performed on the Roche Discovery Ultra. Following deparaffinization and antigen retrieval, a peroxidase block was used. Staining for CD3 (Roche 790-4341), CD19 (Millipore Sigma 119R-18), and CD20 (Roche 760-2531) was performed, followed by secondary antibody incubation, DAB development, and a Hematoxylin counterstain. A pathologist (C.R.) evaluated the slides, which were subsequently scanned under brightfield using the Vectra Polaris. Histological staining and imaging were performed at the University of Michigan Tissue and Molecular Pathology Core.

Spatial sequencing

We utilized the single-cell resolution 10× Xenium platform to conduct spatial transcriptome profiling. Xenium Ranger was used to conduct multi-modal cell segmentation, and Giotto Suite (v.4.2.0) was used for data processing and further quality control. Only transcripts with qv values greater than 10 were used, and cells with at least 30 transcripts were maintained. Symphony was used for cell typing, using cell-type-annotated single-cell RNA sequencing (RNA-seq) data from same tissue as reference. We only utilized cells with ≥75% cell-type assignment rate to retain cells with high-confident cell-type annotations.

DATA AND CODE AVAILABILITY

Data can be made available upon request.

ACKNOWLEDGMENTS

Cabaletta Bio funded the research described herein.

AUTHOR CONTRIBUTIONS

J.R.V., D.N., D.K., and S.B. designed the research; J.S., M.W., Z.V., A.E., J.W., J.C., P.-S.T., and D.K. performed experiments; T.F., C.S., and Q.L. oversaw logistics; C.L., R.T., and D.C. designed the clinical protocol; D.K. and M.G. managed patients; J.F., J.E.G., and P.-S.T. oversaw sample processing; D.N., J.R.V., P.D., L.C.T., Y.C., P.-S. T., J.E.G., D.K., and S.B. analyzed data; T.F., D.N., J.R.V., C.L., D.K., and S.B. drafted the manuscript. All authors reviewed the data and revised the manuscript.

DECLARATION OF INTERESTS

D.N., J.R.V., C.L., T.F., J.S., M.W., Z.V., L.I., A.E., J.W., J.C., S.F., D.K., Q.L., C.S., F.N., D.T., D.B., T.G.R., R.T., G.B., D.C., and S.B. are employees of Cabaletta Bio. J.E.G. has served as an advisor and/or received research support from Almirall, AbbVie, Johnson & Johnson, GSK, Merck, BMS, Novartis, Regeneron, Sanofi, Leo Pharma, and Liferna Therapeutics. M.G. has served as an advisor and/or received support from BMS and Cabaletta Bio. D.K. has served as an advisor and/or received support from AbbVie, BMS, AstraZeneca, Fate Therapeutics, Nkarta, Novartis, and Cabaletta Bio.

SUPPLEMENTAL INFORMATION

Supplemental information can be found online at https://doi.org/10.1016/j.omta.2025.201663.

REFERENCES

1. Ingegnoli, F., Ughi, N., and Mihai, C. (2018). Update on the epidemiology, risk factors, and disease outcomes of systemic sclerosis. Best Pract. Res. Clin. Rheumatol. *32*, 223–240. https://doi.org/10.1016/j.berh.2018.08.005.
2. Sakkas, L.I., Katsiari, C.G., Daoussis, D., and Bogdanos, D.P. (2023). The role of B cells in the pathogenesis of systemic sclerosis: an update. Rheumatology *62*, 1780–1786. https://doi.org/10.1093/rheumatology/keac578.
3. Truchetet, M.E., Brembilla, N.C., and Chizzolini, C. (2023). Current Concepts on the Pathogenesis of Systemic Sclerosis. Clin. Rev. Allergy Immunol. *64*, 262–283. https://doi.org/10.1007/s12016-021-08889-8.
4. Allanore, Y., Simms, R., Distler, O., Trojanowska, M., Pope, J., Denton, C.P., and Varga, J. (2015). Systemic sclerosis. Nat. Rev. Dis. Primers *1*, 15002. https://doi.org/10.1038/nrdp.2015.2.
5. Jaafar, S., Lescoat, A., Huang, S., Gordon, J., Hinchcliff, M., Shah, A.A., Assassi, S., Domsic, R., Bernstein, E.J., Steen, V., et al. (2021). Clinical characteristics, visceral involvement, and mortality in at-risk or early diffuse systemic sclerosis: a longitudinal analysis of an observational prospective multicenter US cohort. Arthritis Res. Ther. *23*, 170. https://doi.org/10.1186/s13075-021-02548-1.
6. Brown, M., and O'Reilly, S. (2019). The immunopathogenesis of fibrosis in systemic sclerosis. Clin. Exp. Immunol. *195*, 310–321. https://doi.org/10.1111/cei.13238.
7. Melissaropoulos, K., Iliopoulos, G., Sakkas, L.I., and Daoussis, D. (2022). Pathogenetic Aspects of Systemic Sclerosis: A View Through the Prism of B Cells. Front. Immunol. *13*, 925741. https://doi.org/10.3389/fimmu.2022.925741.
8. Pattanaik, D., Brown, M., Postlethwaite, B.C., and Postlethwaite, A.E. (2015). Pathogenesis of Systemic Sclerosis. Front. Immunol. *6*, 272. https://doi.org/10.3389/fimmu.2015.00272.
9. Yoshizaki, A., Fukasawa, T., Ebata, S., Yoshizaki-Ogawa, A., and Sato, S. (2022). Involvement of B cells in the development of systemic sclerosis. Front. Immunol. *13*, 938785. https://doi.org/10.3389/fimmu.2022.938785.

10. Ebata, S., Yoshizaki, A., Oba, K., Kashiwabara, K., Ueda, K., Uemura, Y., Watadani, T., Fukasawa, T., Miura, S., Yoshizaki-Ogawa, A., et al. (2021). Safety and efficacy of rituximab in systemic sclerosis (DESIRES): a double-blind, investigator-initiated, randomised, placebo-controlled trial. Lancet. Rheumatol. *3*, e489–e497. https://doi.org/10.1016/S2665-9913(21)00107-7.

11. Maher, T.M., Tudor, V.A., Saunders, P., Gibbons, M.A., Fletcher, S.V., Denton, C.P., Hoyles, R.K., Parfrey, H., Renzoni, E.A., Kokosi, M., et al. (2023). Rituximab versus intravenous cyclophosphamide in patients with connective tissue disease-associated interstitial lung disease in the UK (RECITAL): a double-blind, double-dummy, randomised, controlled, phase 2b trial. Lancet Respir. Med. *11*, 45–54. https://doi.org/10.1016/S2213-2600(22)00359-9.

12. Goswami, R.P., Ray, A., Chatterjee, M., Mukherjee, A., Sircar, G., and Ghosh, P. (2021). Rituximab in the treatment of systemic sclerosis-related interstitial lung disease: a systematic review and meta-analysis. Rheumatology *60*, 557–567. https://doi.org/10.1093/rheumatology/keaa550.

13. Crickx, E., Weill, J.C., Reynaud, C.A., and Mahévas, M. (2020). Anti-CD20-mediated B-cell depletion in autoimmune diseases: successes, failures and future perspectives. Kidney Int. *97*, 885–893. https://doi.org/10.1016/j.kint.2019.12.025.

14. Pollastro, S., Klarenbeek, P.L., Doorenspleet, M.E., van Schaik, B.D.C., Esveldt, R.E.E., Thurlings, R.M., Boumans, M.J.H., Gerlag, D.M., Tak, P.P., Vos, K., et al. (2019). Non-response to rituximab therapy in rheumatoid arthritis is associated with incomplete disruption of the B cell receptor repertoire. Ann. Rheum. Dis. *78*, 1339–1345. https://doi.org/10.1136/annrheumdis-2018-214898.

15. Tur, C., Eckstein, M., Velden, J., Rauber, S., Bergmann, C., Auth, J., Bucci, L., Corte, G., Hagen, M., Wirsching, A., et al. (2025). CD19-CAR T-cell therapy induces deep tissue depletion of B cells. Ann. Rheum. Dis. *84*, 106–114. https://doi.org/10.1136/ard-2024-226142.

16. Auth, J., Müller, F., Völkl, S., Bayerl, N., Distler, J.H.W., Tur, C., Raimondo, M.G., Chenguiti Fakhouri, S., Atzinger, A., Coppers, B., et al. (2025). CD19-targeting CAR T-cell therapy in patients with diffuse systemic sclerosis: a case series. Lancet. Rheumatol. *7*, e83–e93. https://doi.org/10.1016/S2665-9913(24)00282-0.

17. Müller, F., Taubmann, J., Bucci, L., Wilhelm, A., Bergmann, C., Völkl, S., Aigner, M., Rothe, T., Minopoulou, I., Tur, C., et al. (2024). CD19 CAR T-Cell Therapy in Autoimmune Disease - A Case Series with Follow-up. N. Engl. J. Med. *390*, 687–700. https://doi.org/10.1056/NEJMoa2308917.

18. Volkov, J., Nunez, D., Mozaffar, T., Stadanlick, J., Werner, M., Vorndran, Z., Ellis, A., Williams, J., Cicarelli, J., Lam, Q., et al. (2024). Case study of CD19 CAR T therapy in a subject with immune-mediate necrotizing myopathy treated in the RESET-Myositis phase I/II trial. Mol. Ther. *32*, 3821–3828. https://doi.org/10.1016/j.ymthe.2024.09.009.

19. Peng, B.J., Alvarado, A., Cassim, H., Guarneri, S., Wong, S., Willis, J., SantaMaria, J., Martynchuk, A., Stratton, V., Patel, D., et al. (2024). Preclinical specificity & activity of a fully human 41BB-expressing anti-CD19 CART- therapy for treatment-resistant autoimmune disease. Mol. Ther. Methods Clin. Dev. *32*, 101267. https://doi.org/10.1016/j.omtm.2024.101267.

20. Khanna, D., Furst, D.E., Clements, P.J., Allanore, Y., Baron, M., Czirjak, L., Distler, O., Foeldvari, I., Kuwana, M., Matucci-Cerinic, M., et al. (2017). Standardization of the modified Rodnan skin score for use in clinical trials of systemic sclerosis. J. Scleroderma Relat. Disord. *2*, 11–18. https://doi.org/10.5301/jsrd.5000231.

21. Bruce, B., and Fries, J.F. (2003). The Stanford Health Assessment Questionnaire: dimensions and practical applications. Health Qual. Life Outcomes *1*, 20. https://doi.org/10.1186/1477-7525-1-20.

22. Cole, J.C., Khanna, D., Clements, P.J., Seibold, J.R., Tashkin, D.P., Paulus, H.E., Irwin, M.R., Motivala, S.J., and Furst, D.E. (2006). Single-factor scoring validation for the Health Assessment Questionnaire-Disability Index (HAQ-DI) in patients with systemic sclerosis and comparison with early rheumatoid arthritis patients. Qual. Life Res. *15*, 1383–1394. https://doi.org/10.1007/s11136-006-0018-8.

23. Khanna, D., Huang, S., Lin, C.J.F., and Spino, C. (2021). New composite endpoint in early diffuse cutaneous systemic sclerosis: revisiting the provisional American College of Rheumatology Composite Response Index in Systemic Sclerosis. Ann. Rheum. Dis. *80*, 641–650. https://doi.org/10.1136/annrheumdis-2020-219100.

24. Bergmann, C., Müller, F., Distler, J.H.W., Györfi, A.H., Völkl, S., Aigner, M., Kretschmann, S., Reimann, H., Harrer, T., Bayerl, N., et al. (2023). Treatment of a patient with severe systemic sclerosis (SSc) using CD19-targeted CAR T cells. Ann. Rheum. Dis. *82*, 1117–1120. https://doi.org/10.1136/ard-2023-223952.

25. Bergmann, C., Müller, F., Auth, J., Taubmann, J., Aigner, M., Werner, D., Wirsching, A., Knitza, J., Gyorfi, H., Distler, J., et al. (2023). 6 months-follow up data of systemic sclerosis patients treated with CD 19 targeting CAR-T cells. Arthritis Rheumatol. *75*, 5181–5182. https://acrabstracts.org/abstract/6-months-follow-up-data-of-systemic-sclerosis-patients-treated-with-cd-19-targeting-car-t-cells/.

26. Kowal-Bielecka, O., Fransen, J., Avouac, J., Becker, M., Kulak, A., Allanore, Y., Distler, O., Clements, P., Cutolo, M., Czirjak, L., et al. (2017). Update of EULAR recommendations for the treatment of systemic sclerosis. Ann. Rheum. Dis. *76*, 1327–1339. https://doi.org/10.1136/annrheumdis-2016-209909.

27. Elhai, M., Meune, C., Avouac, J., Kahan, A., and Allanore, Y. (2012). Trends in mortality in patients with systemic sclerosis over 40 years: a systematic review and meta-analysis of cohort studies. Rheumatology *51*, 1017–1026. https://doi.org/10.1093/rheumatology/ker269.

28. Sullivan, K.M., Goldmuntz, E.A., Keyes-Elstein, L., McSweeney, P.A., Pinckney, A., Welch, B., Mayes, M.D., Nash, R.A., Crofford, L.J., Eggleston, B., et al. (2018). Myeloablative Autologous Stem-Cell Transplantation for Severe Scleroderma. N. Engl. J. Med. *378*, 35–47. https://doi.org/10.1056/nejmoa1703327.

29. Nunez, D., Patel, D., Volkov, J., Wong, S., Vorndran, Z., Müller, F., Aigner, M., Völkl, S., Mackensen, A., Schett, G., and Basu, S. (2023). Cytokine and reactivity profiles in SLE patients following anti-CD19 CART therapy. Mol. Ther. Methods Clin. Dev. *31*, 101104. https://doi.org/10.1016/j.omtm.2023.08.023.

30. Giordano, D., Kuley, R., Draves, K.E., Roe, K., Holder, U., Giltiay, N.V., and Clark, E.A. (2020). BAFF Produced by Neutrophils and Dendritic Cells Is Regulated Differently and Has Distinct Roles in Antibody Responses and Protective Immunity against West Nile Virus. J. Immunol. *204*, 1508–1520. https://doi.org/10.4049/jimmunol.1901120.

31. Furie, R.A., Aroca, G., Cascino, M.D., Garg, J.P., Rovin, B.H., Alvarez, A., Fragoso-Loyo, H., Zuta-Santillan, E., Schindler, T., Brunetta, P., et al. (2022). B-cell depletion with obinutuzumab for the treatment of proliferative lupus nephritis: a randomised, double-blind, placebo-controlled trial. Ann. Rheum. Dis. *81*, 100–107. https://doi.org/10.1136/annrheumdis-2021-220920.

32. Ochfeld, E., Hans, V., Marin, W., Ahsan, N., Morgan, G., Pachman, L.M., and Khojah, A. (2022). Coding joint: kappa-deleting recombination excision circle ratio and B cell activating factor level: predicting juvenile dermatomyositis rituximab response, a proof-of-concept study. BMC Rheumatol. *6*, 36. https://doi.org/10.1186/s41927-022-00265-z.

33. Lavie, F., Miceli-Richard, C., Ittah, M., Sellam, J., Gottenberg, J.E., and Mariette, X. (2007). Increase of B cell-activating factor of the TNF family (BAFF) after rituximab treatment: insights into a new regulating system of BAFF production. Ann. Rheum. Dis. *66*, 700–703. https://doi.org/10.1136/ard.2006.060772.

34. Bosello, S., De Santis, M., Lama, G., Spanò, C., Angelucci, C., Tolusso, B., Sica, G., and Ferraccioli, G. (2010). B cell depletion in diffuse progressive systemic sclerosis: safety, skin score modification and IL-6 modulation in an up to thirty-six months follow-up open-label trial. Arthritis Res. Ther. *12*, R54. https://doi.org/10.1186/ar2965.

35. Sanz, I., Wei, C., Jenks, S.A., Cashman, K.S., Tipton, C., Woodruff, M.C., Hom, J., and Lee, F.E.H. (2019). Challenges and Opportunities for Consistent Classification of Human B Cell and Plasma Cell Populations. Front. Immunol. *10*, 2458. https://doi.org/10.3389/fimmu.2019.02458.

36. Marie-Cardine, A., Divay, F., Dutot, I., Green, A., Perdrix, A., Boyer, O., Contentin, N., Tilly, H., Tron, F., Vannier, J.P., and Jacquot, S. (2008). Transitional B cells in humans: characterization and insight from B lymphocyte reconstitution after hematopoietic stem cell transplantation. Clin. Immunol. *127*, 14–25. https://doi.org/10.1016/j.clim.2007.11.013.

37. Taubmann, J., Müller, F., Boeltz, S., Knitza, J., Völkl, S., Aigner, M., Kleyer, A., Minnopoulou, I., Locatelli, F., D'agostino, M.A., et al. (2023). First evidence for efficacy of CAR T cell treatment in refractory antisynthetase syndrome [abstract]. Ann. Rheum. Dis. *82*, 957–958. https://doi.org/10.1136/annrheumdis-2023-eular.3842.

38. Khanna, D., Denton, C.P., Assassi, S., Kuwana, M., Allanore, Y., Domsic, R.T., Kleoudis, C., Xu, J., Csomor, E., Seo, C., et al. (2024). A randomised, parallel-group, double-blind, placebo-controlled phase 3 study to Determine the effectiveness of the

type I interferon receptor antibody, Anifrolumab, In SYstemic sclerosis: DAISY study design and rationale. Clin. Exp. Rheumatol. *42*, 1635–1644. https://doi.org/10.55563/clinexprheumatol/s8qcyu.

39. Pabst, T., Joncourt, R., Shumilov, E., Heini, A., Wiedemann, G., Legros, M., Seipel, K., Schild, C., Jalowiec, K., Mansouri Taleghani, B., et al. (2020). Analysis of IL-6 serum levels and CAR T cell-specific digital PCR in the context of cytokine release syndrome. Exp. Hematol. *88*, 7–14.e3. https://doi.org/10.1016/j.exphem.2020.07.003.

40. Mackensen, A., Müller, F., Mougiakakos, D., Böltz, S., Wilhelm, A., Aigner, M., Völkl, S., Simon, D., Kleyer, A., Munoz, L., et al. (2022). Anti-CD19 CAR T cell therapy for refractory systemic lupus erythematosus. Nat. Med. *28*, 2124–2132. https://doi.org/10.1038/s41591-022-02017-5.

41. Ross, L., Nikpour, M., D'Aoust, J., Khanna, D., Merkel, P.A., Pauling, J.D., and Baron, M. (2023). Patient and Physician Global Assessments of Disease Status in Systemic Sclerosis. Arthritis Care Res. *75*, 1443–1451. https://doi.org/10.1002/acr.25056.

42. Schieir, O., Thombs, B.D., Hudson, M., Boivin, J.F., Steele, R., Bernatsky, S., Hanley, J., and Baron, M.; Canadian Scleroderma Research Group (2010). Prevalence, severity, and clinical correlates of pain in patients with systemic sclerosis. Arthritis Care Res. *62*, 409–417. https://doi.org/10.1002/acr.20108.

43. Yellen, S.B., Cella, D.F., Webster, K., Blendowski, C., and Kaplan, E. (1997). Measuring fatigue and other anemia-related symptoms with the Functional Assessment of Cancer Therapy (FACT) measurement system. J. Pain Symptom Manage. *13*, 63–74. https://doi.org/10.1016/s0885-3924(96)00274-6.

44. Hasseli-Frabel, R., Hermann, W., Sander, O., and Triantafyllias, K. (2022). [Nailfold capillaroscopy-Principles and clinical application]. Z. Rheumatol. *81*, 313–322. https://doi.org/10.1007/s00393-022-01200-w.

45. Smith, V., Herrick, A.L., Ingegnoli, F., Damjanov, N., De Angelis, R., Denton, C.P., Distler, O., Espejo, K., Foeldvari, I., Frech, T., et al. (2020). Standardisation of nailfold capillaroscopy for the assessment of patients with Raynaud's phenomenon and systemic sclerosis. Autoimmun. Rev. *19*, 102458. https://doi.org/10.1016/j.autrev.2020.102458.

46. Kalos, M., Levine, B.L., Porter, D.L., Katz, S., Grupp, S.A., Bagg, A., and June, C.H. (2011). T cells with chimeric antigen receptors have potent antitumor effects and can establish memory in patients with advanced leukemia. Sci. Transl. Med. *3*, 95ra73. https://doi.org/10.1126/scitranslmed.3002842.

47. Milbury, C.A., Zhong, Q., Lin, J., Williams, M., Olson, J., Link, D.R., and Hutchison, B. (2014). Determining lower limits of detection of digital PCR assays for cancer-related gene mutations. Biomol. Detect. Quantif. *1*, 8–22. https://doi.org/10.1016/j.bdq.2014.08.001.

48. Sugimoto, H., Chen, S., Minembe, J.P., Chouitar, J., He, X., Wang, H., Fang, X., and Qian, M.G. (2021). Insights on Droplet Digital PCR-Based Cellular Kinetics and Biodistribution Assay Support for CAR-T Cell Therapy. AAPS J. *23*, 36. https://doi.org/10.1208/s12248-021-00560-6.

OMTA, Volume *34*

Supplemental information

Case study of resecabtagene autoleucel in a subject with diffuse cutaneous systemic sclerosis treated in the RESET-SSc trial

Daniel Nunez, Jenell R. Volkov, Courtney Little, Monalisa Ghosh, Pei-Suen Tsou, Thomas Furmanak, Poulami Dey, Lam C. Tsoi, Carleigh Zahn, Rachael Bogle, Yuli Cai, Jennifer Fox, Jason Stadanlick, Mallorie Werner, Zachary Vorndran, Larissa Ishikawa, Alexandra Ellis, Jazmean Williams, Justin Cicarelli, Steve Flannagan, Danielle Kobulsky, Quynh Lam, Chris Schmitt, Fatemeh Nezhad, Daniel Thompson, Dominick Braccia, Tania Gonzalez Rivera, Raj Tummala, Johann E. Gudjonsson, Charles Ross, Gwendolyn Binder, David Chang, Samik Basu, and Dinesh Khanna

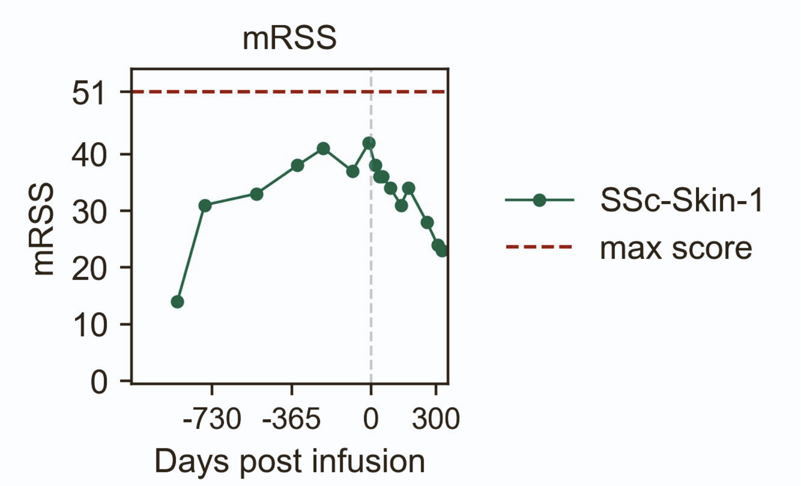

Figure S1. Historical mRSS from patient prior to rese-cel infusion and for up to 36 weeks post-infusion. Dashed vertical line represents infusion of rese-cel. X-axis is time from infusion. Red horizontal dashed line represents maximum possible mRSS.

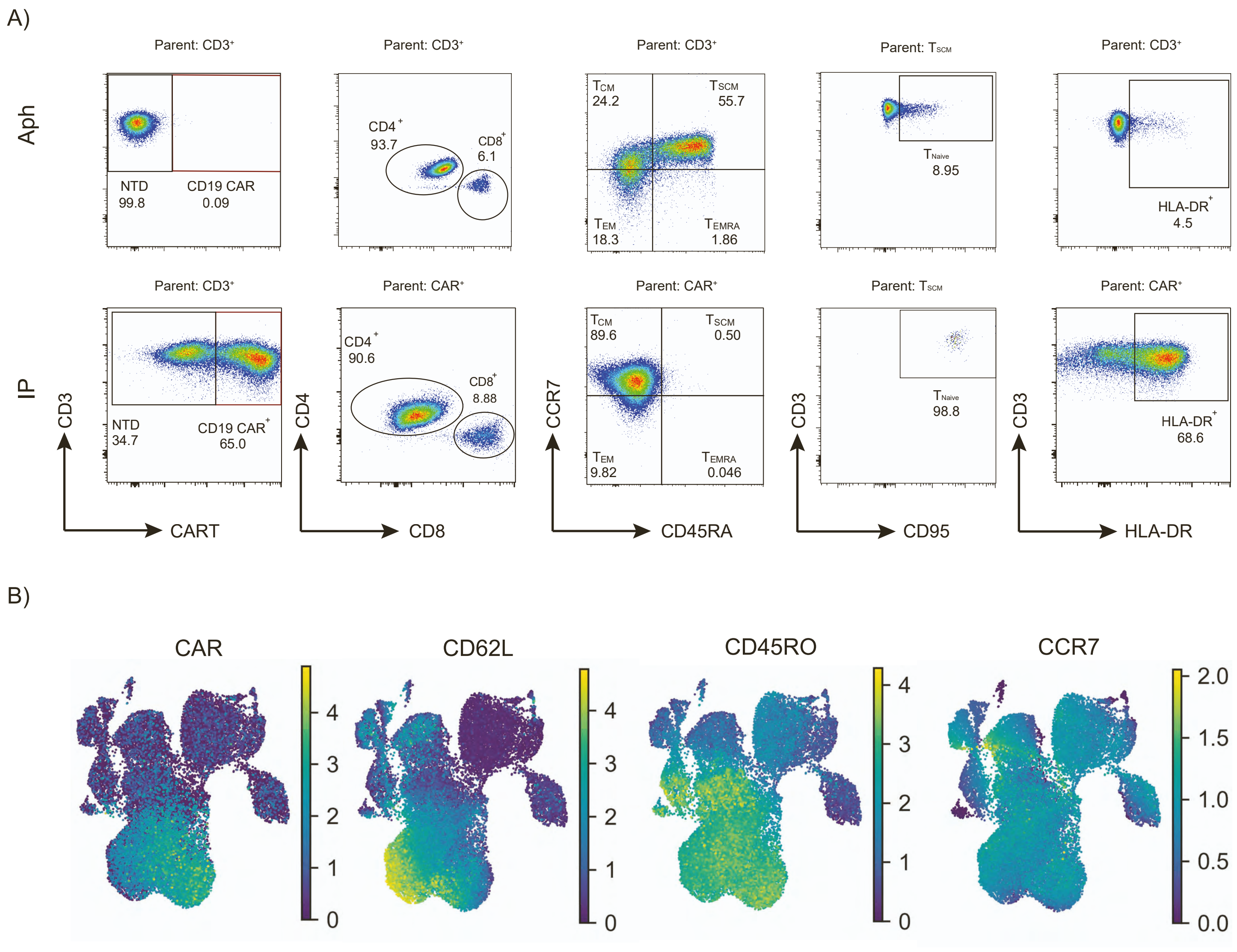

Figure S2. (A) Flow cytometry plots from patient's apheresis (Aph) and infusion product (IP). From left to right: the percentage of $CD3^+$ T cells that are CAR^+, the prorption of T cells (for Aph) and of CAR^+ T cells (for IP) that are $CD4^+$ and $CD8^+$, the memory phenotype of T cells (for Aph) and of CAR^+ T cells (for IP) via CCR7 and CD45RA labeling, the CD95 expression within the T_{SCM} parent gate, and the percentage $HLA\text{-}DR^+$ T cells (for Aph) and of CAR^+ T cells (for IP). **(B)** Single-cell sequencing from patient's Aph and IP. CAR expression is shown as the natural logarithm of the CAR mRNA count divided by the total RNA counts per cell. CD62L, CD45RO, and CCR7 expression is shown as the natural logarithm of each protein count divided by the total portein counts per cell.

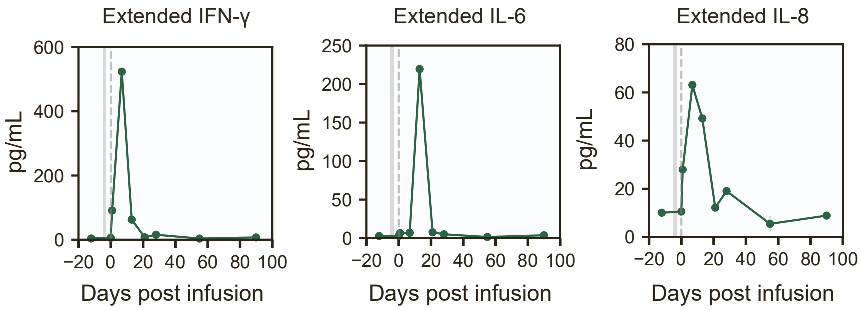

Figure S3. Extended serum cytokine profiling from patient SSc-Skin-1 over the first 12 weeks following rese-cel infusion. From left to right, plots depict serum concentrations of IFN-γ, IL-6, and IL-8 in pg/mL. The gray vertical dotted line indicates the day of infusion and the gray vertical shading indicates the period in time when the patient underwent preconditioning.
